# Coexisting myasthenia gravis, myositis, and polyneuropathy induced by ipilimumab and nivolumab in a patient with non-small-cell lung cancer: A case report and literature review

**DOI:** 10.1097/MD.0000000000009262

**Published:** 2017-12-15

**Authors:** Jia-Hung Chen, Kang-Yun Lee, Chaur-Jong Hu, Chen-Chih Chung

**Affiliations:** a Department of Neurology; b Division of Pulmonary Medicine, Department of Internal Medicine, Shuang Ho Hospital, Taipei Medical University, New Taipei City; c Department of Neurology, School of Medicine, Taipei Medical University, Taipei, Taiwan.

**Keywords:** checkpoint inhibitors, immune-related adverse events, myasthenia gravis, myositis, polyneuropathy

## Abstract

**Rationale:**

Immune checkpoint inhibitors have led to the development of new approaches for cancer treatment with positive outcomes. However, checkpoint blockade is associated with a unique spectrum of immune-related adverse events (irAEs), which may cause irreversible neurological deficits and even death.

**Patient concerns:**

We presented a case of a 57-year-old man with non-small-cell lung cancer.who developed ptosis, dyspnea, and muscle weakness as initial symptoms with progression after the treatment with ipilimumab and nivolumab.

**Diagnoses:**

Myasthenia gravis was confirmed by serum acetylcholine receptor antibody and single fiber electromyography. Myositis was identified by high level of serum creatine phosphokinase and electromyography. Polyneuropathy was identified by nerve conduction study.

**Interventions:**

The patient underwent treatment with steroid and pyridostigmine. Respiratory rehabilitation was also performed.

**Outcomes:**

Dyspnea and muscle weakness improved gradually. Ipilimumab and nivolumab were permanently discontinued.

**Lessons:**

This case has increased the clinical awareness by indicating that the checkpoint inhibitors-related neurological irAEs could be complicated and simultaneously involve multiple neurological systems. Early recognition and complete evaluation are critical in clinical practice.

## 1 Introduction

In recent years, advances in the understanding of the regulatory mechanisms of the immune system have led to the development of new approaches for cancer treatment. Immune checkpoint inhibitors are the first successful examples of such approaches. Several agents that target cytotoxic T-lymphocyte antigen-4 (CTLA-4), programmed cell death-1 (PD-1), and programmed deathligand 1 (PD-L1) inhibitors have been introduced for various oncological conditions. Ipilimumab, a fully humanized monoclonal antibody that directly inhibits the function of the immune checkpoint inhibitor CTLA-4, has been shown to improve survival in patients with metastatic cancers.^[1]^ Nivolumab, which targets the immune checkpoint inhibitor PD-1, has also been used in various cancers including non-small-cell lung cancer (NSCLC). Combination therapy with ipilimumab and nivolumab has been studied in patients with NSCLC and showed encouraging results.^[2]^ However, checkpoint blockade is associated with a unique spectrum of immune-related adverse events (irAEs). Various neurological irAEs involving the central and peripheral nervous systems have been reported.^[3]^ In this report, we describe a patient who developed myasthenia gravis, myositis, and polyneuropathy after treatment with ipilimumab and nivolumab for NSCLC. Patient consent was not given since the patient had died, but Institutional Review Board had approved this case report. (TMUJIRB N201706025).

## 2 Case presentation

A 57-year-old man experienced difficulty in swallowing, hoarseness, and presence of bloody sputum for half a month. Laryngoscopy revealed right vocal cord palsy. Chest computed tomography (CT) showed a 3.9-cm irregular mass at the right apical lung with several ground-glass opacity lesions in bilateral lung fields. CT-guided biopsy confirmed the histopathological diagnosis of squamous cell carcinoma. Immunohistochemistry was positive for cytokeratin (CK)-7, CK-5/6, and deltaNp63 (p40), and it was negative for CK20, TTF-1, napsinA, and CD56. A bone scan revealed bony metastases in the left fourth rib and the right iliac bone. Brain magnetic resonance imaging did not show brain metastasis. Therefore, a diagnosis of stage IV squamous cell carcinoma was established.

The patient received immune checkpoint therapy consisting of nivolumab (3 mg/kg every 2 weeks) and ipilimumab (1 mg/kg every 6 weeks). After the first cycle of ipilimumab and the second cycle of nivolumab, elevated liver function test values were noted (glutamic-oxalocetic transaminase [GOT] = 169 IU/L [reference range, 5–40 IU/L] and glutamic pyruvic transaminase [GPT] = 148 IU/L [reference range, 5–40 IU/L]). Two weeks later, the patient gradually developed symptoms such as drooping of eyelids, dropped head, limb weakness, unsteadiness in walking, and mild dyspnea. Physical and neurological examination showed ptosis of the right eye and weakness of the neck extensor muscles and proximal limb muscles (Medical Research Council [MRC] scale for muscle strength = 4). In addition, weakness of respiratory muscles with dyspnea was observed. Significant muscle atrophy over the extremities and deep tendon hyporeflexia were observed. Serum biochemistry examination showed elevated levels of creatine phosphokinase (CPK, 2682 U/L [reference range, 38–397 U/L]) and acetylcholine receptor (AchR) antibody (0.7 nmol/L [reference range <0.5 nmol/L]). Cerebrospinal fluid examination revealed a slightly lower protein level at 13 mg/dL (reference range, 15–45 mg/dL) without pleocytosis (WBC count, 0/μL [reference ranges: WBC, 0–5/μL; neutrophil, 16%; and lymphocyte, 0%]). Nerve conduction study showed sensorimotor polyneuropathy of axonal degeneration, and electromyography (EMG) showed active denervation and myopathic changes in the sampled muscles (Table 1). Repetitive stimulation test (RST) with 3 Hz stimulation over median, accessory, and facial nerve did not show decremental responses. Single-fiber EMG over the right orbicularis oculi demonstrated a mean consecutive difference of 74 μs (reference range <50 μs). On the basis of these findings, a diagnosis of the following 3 concurrent disorders of the peripheral nervous system was made: myasthenia gravis, myositis, and sensorimotor polyneuropathy.

**Table 1:**
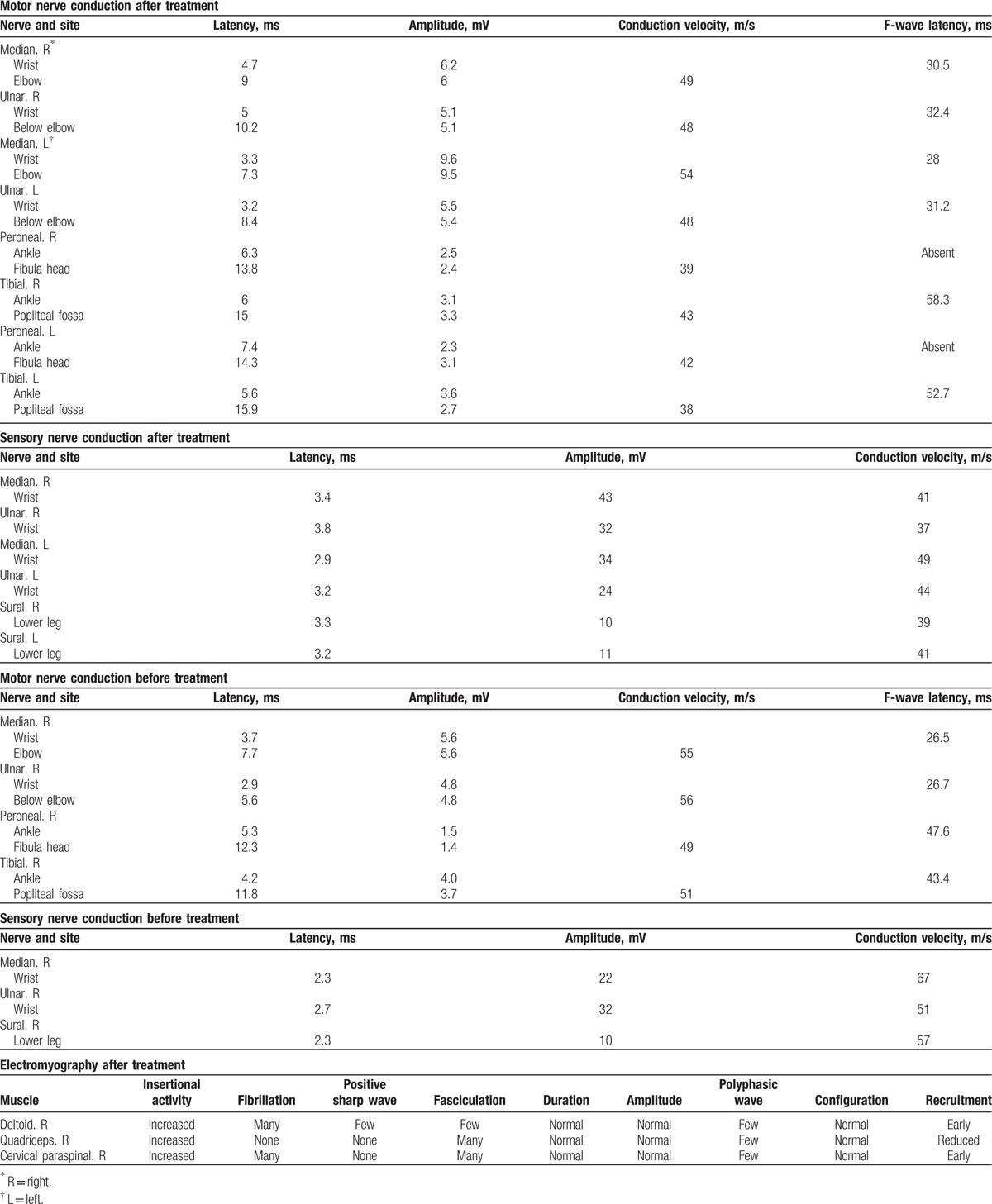
Nerve conduction study and electromyography after and before immune checkpoints inhibitors treatment.

The patient underwent treatment with intravenous prednisolone (2 mg/kg/d for 5 days followed by 1 mg/kg/d for 2 days) and oral pyridostigmine (60 mg 3 times a day). His CPK level decreased and reached the normal level (133 U/L) after the treatment. His symptoms improved gradually, and he could tilt up his head and walk steadily for a short distance. The muscle strength of the extremities also improved (MRC = 4+), and dyspnea subsided. The patient continued taking oral prednisolone (1 mg/kg/d) and pyridostigmine (60 mg 3 times a day) for maintenance and immunomodulatory therapy for myasthenia gravis, myositis, and polyneuropathy, in addition to receiving respiratory rehabilitation. However, the patient developed hospital-acquired pneumonia 1 week later and subsequently died of severe sepsis.

## 3 Discussion

In this report, we present a patient who developed coexisting myasthenia gravis, myositis, and polyneuropathy after treatment with ipilimumab and nivolumab for NSCLC. To the best of our knowledge, this is the first case report of severe neurological adverse effects involving all neuromuscular junctions, muscles, and nerves related to the therapy of checkpoint inhibitors for lung cancer.

Immune checkpoint blockade has increased our understanding of complex interactions between the immune system and cancer cells. These therapies have shown favorable outcomes in patients with cancer. However, a broad spectrum of adverse events has been reported for almost every body system, and this may limit the clinical application of these therapies. Among these adverse events, neurological complications were sometimes difficult to recognize and may lead to fatal outcomes.

Myasthenia gravis is an autoimmune disorder in which an antibody-mediated, T-cell-dependent immunological attack is directed at proteins in the postsynaptic membrane of the neuromuscular junction. In severe cases, myasthenia gravis may cause respiratory failure and death. Several case reports of myasthenia gravis caused by immunotherapy with either ipilimumab, nivolumab, or both have been reported (Table 2). All these patients developed symptoms after 1 to 3 doses of immunotherapy, and most of them had an elevated AchR antibody level. These patients were treated with pyridostigmine, steroid, plasma exchange, or intravenous immunoglobulin (IVIG), and the symptoms of most patients resolved after adequate management.

**Table 2:**
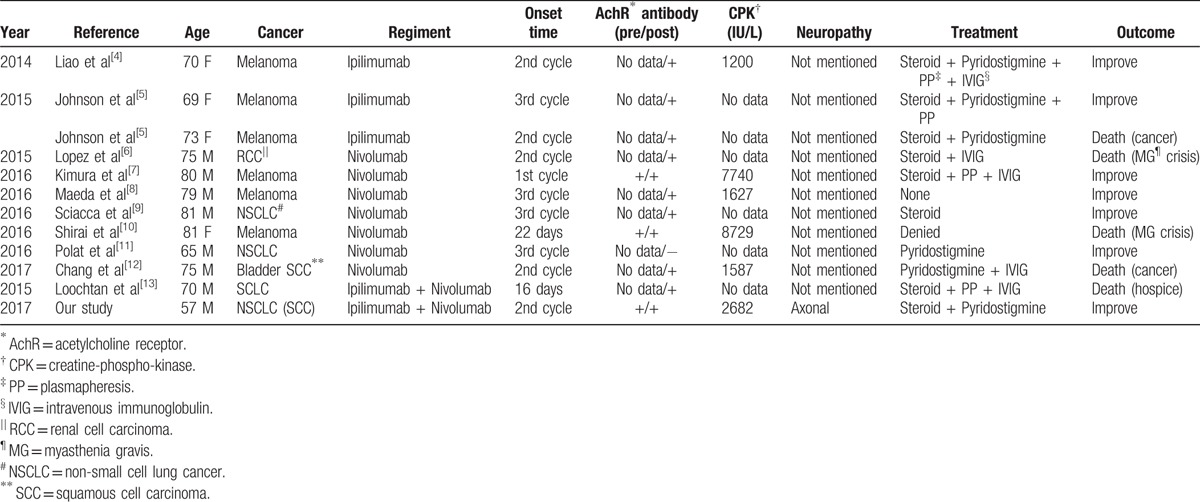
Reported case of myasthenia gravis, myositis, and polyneuropathy caused by immune checkpoints inhibitors.

Polymyositis, another autoimmune disorder of cellular immunity, is most commonly associated with other systemic autoimmune diseases. Studies have demonstrated a CD8 T-cell-mediated cytotoxic process directed against unidentified muscle antigens. Only 2 case reports of polymyositis caused by immunotherapy have been reported thus far. In one case, polymyositis was induced by combination therapy with ipilimumab and nivolumab in a patient with metastatic urothelial carcinoma^[14]^ and in the other case, polymyositis and myasthenia gravis were caused after treatment with nivolumab in a patient with malignant melanoma.^[7]^ In both cases, the adverse event developed after the first or second dose of immunotherapy.

Polyneuropathy refers to a generalized and homogeneous disease process affecting peripheral nerves, with the distal parts being affected more prominently. Immune-mediated polyneuropathies are divided into acute and chronic forms. Both have been described as complications of ipilimumab and, to a lesser extent, of nivolumab. The incidence of polyneuropathy is rare, occurring in <1% of patients. Most cases are mild (grade 1–2); however, fatal cases have been reported.^[15]^

Dropped head and proximal muscle weakness could be noted in patients with myasthenia gravis, myositis, or polyneuropathy. Our patient developed complications involving all these 3 nervous diseases, which may have concurrently aggravated his weakness and made it impractical to distinguish the disorder that contributed more to his symptoms. Unlike typical myasthenia gravis, the severity and outcome of immune checkpoint inhibitor-associated myasthenia gravis did not appear to be correlated with their AchR titers (Table 2). Most of these reported cases did not have pretreatment AchR antibody data. However, almost all patients developed Ach antibodies after the presentation of symptoms. Similar to our patient, half of the previously reported cases had an elevated CPK level, which may be due to rhabdomyolysis or myositis. However, whether these patients had subclinical autoimmune diseases before receiving checkpoint inhibitors or all these immune disorders were activated in different ways, as we recognized these diseases in our patient, remains unclear and warrants further research.

Currently, the management of these irAEs is mostly based on clinical experience. A review suggested corticosteroids with 0.5 to 1 mg/kg/d for grade 2 irAEs and 1–2 mg/kg/d for grade 3–4 irAEs an initial treatment.^[3]^ Further immunosuppressive therapy with IVIG, plasma exchange, or a monoclonal antibody should be considered under the situation of severe neurological adverse events that do not respond to steroids. Skipping a dose of immunotherapy or discontinuing immunotherapy can be considered depending on the benefit/risk ratio of each given situation.

Although immune checkpoint blockade is typically described as being well tolerated, it still generates life-threatening irAEs that are sometimes severe and irreversible. This case has increased clinical awareness by indicating that the neurological irAEs of checkpoint inhibitors could simultaneously involve multiple neurological systems. Early recognition of aberrant immune activation and complete evaluation of the entire nervous system upon the occurrence of irAEs are critical for clinical practice in the treatment with immune checkpoint inhibitors.

## Acknowledgments

The authors thank Paula Bensley and Yankz from Wallace Academic Editing for editing this manuscript.
